# Normal Striatal Vesicular Acetylcholine Transporter Expression in Tourette Syndrome

**DOI:** 10.1523/ENEURO.0178-17.2017

**Published:** 2017-08-07

**Authors:** Roger L. Albin, Christine Minderovic, Robert A. Koeppe

**Affiliations:** 1 Neurology Service and Geriatrics Research, Education, and Clinical Center, Veterans Affairs Ann Arbor Health System, Ann Arbor, MI 48105; 2 Department of Neurology, University of Michigan, Ann Arbor, MI 48109; 3 University of Michigan Morris K. Udall Center, Ann Arbor, MI 41809; 4 Department of Radiology, University of Michigan, Ann Arbor, MI 48109

**Keywords:** Acetylcholine, Striatum, Tourette Syndrome, VChAT

## Abstract

Considerable prior work suggests basal ganglia dysfunction in Tourette syndrome (TS). Analysis of a small number of postmortem specimens suggests deficits of some striatal interneuron populations, including striatal cholinergic interneurons. To assess the integrity of striatal cholinergic interneurons in TS, we used [^18^F]FEOBV positron emission tomography (PET) to quantify striatal vesicular acetylcholine transporter (VAChT) expression, a measure of cholinergic terminal density, in human TS and control subjects. We found no evidence of striatal cholinergic deficits. Discrepant imaging and postmortem analysis results may reflect agonal or postmortem changes, medication effects, or significant disease heterogeneity.

## Significance Statement

Prior postmortem analyses suggest loss of striatal cholinergic interneurons in Tourette syndrome (TS). We evaluated this possibility with [^18^F]FEOBV positron emission tomography (PET), a ligand for the vesicular acetylcholine transporter (VAChT) and measure of cholinergic terminal integrity. We studied TS and matched control subjects. There were no differences between TS and control subjects in striatal [^18^F]FEOBV binding, consistent with intact striatal cholinergic neurons in TS.

## Introduction

Tourette syndrome (TS) is a common neurodevelopmental disorder characterized by motor and phonic tics (Robertson, 2012). TS is associated often with comorbid behavioral problems, notably obsessive-compulsive behaviors, and attention deficit-hyperactivity disorder. Considerable evidence points to basal ganglia dysfunction in TS (Albin and Mink, 2006; Ganos et al., 2013). Striatal dysfunction is suggested by response of tics to dopamine antagonists and the presence of tics in other disorders, such as Huntington disease, with unequivocal striatal pathology. Tics are often conceptualized as disordered habits, and habit formation and maintenance are thought to be primary functions of basal ganglia circuits (Yin and Knowlton, 2006; Delorme et al., 2016). Accumulated structural and functional imaging evidence strongly supports the concept of basal ganglia, and particularly striatal, dysfunction in TS (Ganos et al., 2013).

Pathologic evidence of basal ganglia and striatal abnormalities, however, is sparse, as only a small number of postmortem specimens have been analyzed. Vaccarino and colleagues reported several findings in anatomic analyses of a small number (*N* = 5) of postmortem TS specimens (Kalanithi et al., 2005; Kataoka et al., 2010). This group reported diminished striatal parvalbumin-immunoreactive interneurons (probable fast spiking interneurons) and dorsal striatal cholinergic interneurons. In addition, this group reported abnormal distribution of pallidal parvalbumin-immunoreactive neurons (probable GABAergic projection neurons), suggesting abnormal neuronal migration in TS. Subsequent transcriptomic analysis of a larger set (*N* = 9) of postmortem TS striata also suggested diminished striatal cholinergic interneurons (Lennington et al., 2016).

The reported deficit of dorsal striatal cholinergic interneurons is substantial, ∼50%, and diminished cholinergic signaling is a plausible contributor to TS pathophysiology. While only a small fraction (∼1%) of striatal neurons, cholinergic interneurons ramify widely within the striatum and play important roles in modulating striatal functions (Deffains and Bergman, 2015). Xu et al. (2015) reported that mice with selective depletion of dorsolateral striatal interneurons exhibited motor behaviors analogous to tics.

New molecular imaging methods allow quantification of striatal cholinergic terminals *in vivo*. [^18^F]FEOBV is a ligand for the vesicular acetylcholine transporter (VAChT), the protein responsible for pumping acetylcholine into synaptic vesicles. VAChT is uniquely expressed by cholinergic neurons and [^18^F]FEOBV positron emission tomography (PET) effectively cholinergic delineates projection systems *in vivo* (Petrou et al., 2014). To determine if the postmortem results reported previously reflect the integrity of striatal cholinergic interneurons in TS, we used [^18^F]FEOBV PET to measure striatal cholinergic terminal density in TS and control subjects.

## Materials and Methods

### Subjects and subject characterization

This study was approved by the University of Michigan Medical School Institutional Review Board. TS and control subjects between the ages of 18 and 46 were recruited for study participation. Informed consent was obtained from all subjects. TS was defined using conventional DSM criteria. Exclusion criteria included history of substance abuse, history of neurologic disease (other than TS) and for control subjects, history of significant psychiatric disease. Potential subjects using medications that might interact with cholinergic neurotransmission were excluded. All subjects underwent a standard evaluation including recording of demographic information, medical history, concomitant medications, history of TS, general physical and neurologic examination, the Yale Global Tourette Severity Scale (YGTSS), the Yale-Brown Obsessive Compulsive Scale (YOBCS), and the Connors’ Adult ADHD Rating Scale (self-administered short form, CAARS-S:S). All subjects had normal neurologic examinations (other than presence of tics) on the days of evaluations. We studied 18 men and 4 women.

### PET methods

[^18^F]FEOBV was prepared as described previously (Shao et al., 2011; Petrou et al., 2014). [^18^F]FEOBV (288-318 MBq) was administered via intravenous bolus. Subjects were scanned on an ECAT Exact HR+ PET tomograph (Siemens Molecular Imaging). Brain imaging was conducted in three imaging periods. The first period began at injection and continued for 90 min. The subjects were then given a 30-min break, followed by a second imaging period from 120–150 min, an additional break, and then a third imaging period from 180–210 min. Dynamic PET scans were reconstructed using Fourier rebinning (FORE) and the iterative 2D-OSEM algorithm, with four iterations, 16 subsets, and no postreconstruction smoothing. The entire dynamic sequence was coregistered to the final form of the first 90-min scanning period.

The primary analysis was performed using the average of the coregistered 120- to 150- and 180- to 210-min scan periods (termed “late static”). FreeSurfer was used on each individual’s T1-weighted MR to define gray matter and white matter voxels. The entire coregistered PET sequence was registered to the MR. The segmented white matter mask from FreeSurfer (1 for WM; 0 elsewhere) was smoothed to PET resolution, and then a threshold of 0.90 applied to the mask, yielding a volume of interest where the partial volume contribution from non-white matter regions to the mask was <10% for every voxel. This white matter volume-of-interest was then applied to the PET data and used as a normalizing (intensity scaling) factor for the late static scan.

Besides the standard static analysis, we applied two different compartmental kinetic modeling techniques to the entire dynamic sequence, one using metabolite-corrected arterial blood sampling and a full two-tissue compartment model, and a second using reference tissue model with subcortical white matter as the reference tissue (Logan et al., 1996). The results using all three approaches were yielded very similar results and the same conclusions, so only the late static scan analysis is presented.

Right and left caudate and putamen [^18^F]FEOBV binding values were averaged to produce to produce single caudate and putaminal values for each subject.

MRI methods: MRI imaging was performed on a 3 Tesla Philips Ingenia System (Philips) using a 15-channel head coil. A standard T1 weighted series of a 3D inversion recovery prepared turbo-field echo was performed in the sagittal plane using TR/TE/TI = 9.8/4.6/1041 ms; turbo factor = 200; single average; FOV = 240 × 200 × 160 mm; acquired matrix = 240 × 200. One hundred and sixty slices were reconstructed to 1-mm isotropic resolution. Cortical and Subcortical segmentation was performed with FreeSurfer version 5 (freesurfer-i686-apple-darwin9.8.0-stable5-20110525; https://surfer.nmr.mgh.harvard.edu/fswiki/FreeSurferMethodsCitation), developed by the Laboratory for Computational Neuroimaging at the Athinoula A. Martinos Center for Biomedical Imaging. The programs were run in the fully automated mode. Volumes for all standard FreeSurfer volumes were obtained.

### Statistical analysis

Striatal [^18^F]FEOBV binding in TS and NC subjects was compared with two-tailed *t* tests without correction for multiple comparisons. Ages of TS and NC subjects were compared with a two-tailed *t* test. Striatal volumes were compared with two-tailed *t* tests. YBOCS and CAARS-S:S results in TS and NC subjects were compared with non-directional Mann–Whitney *U* tests. Potential correlation between global YGTSS scores and striatal [^18^F]FEOBV binding were assessed with Pearson product moment correlation analysis.

## Results

### Subject characteristics

We studied 12 TS subjects (2 women:10 men) and 10 control subjects (2 women:8 men). Clinical Characteristics are presented in Table 1.

**Table 1.**
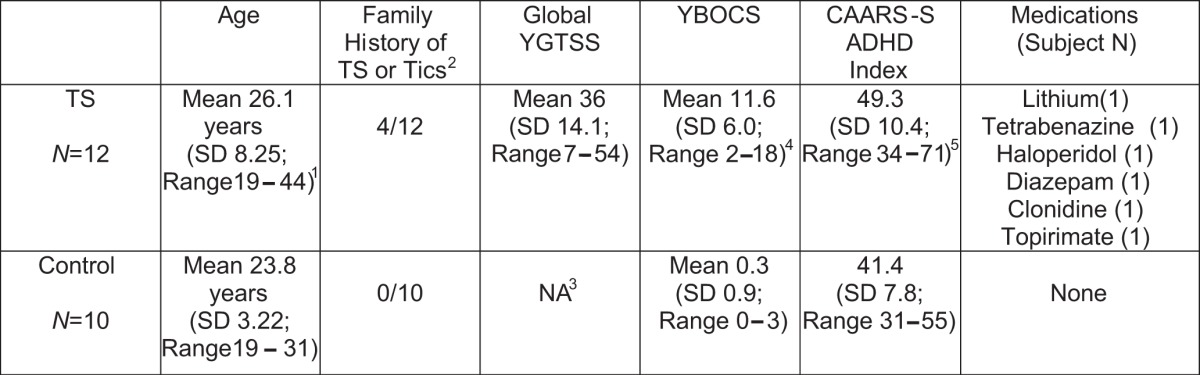
Clinical characteristics of subjects

^1^ TS not different from NC; *p* = 0.42; two-tailed *t* test.

^2^ Possible or definite TS/Tics.

^3^ Not applicable.

^4^ TS different from NC; *p* < 0.0001; nondirectional Mann–Whitney *U* test.

^5^ TS different from NC; *p* < 0.05; nondirectional Mann–Whitney *U* test.

### PET results

There was no difference in striatal [^18^F]FEOBV binding between TS and NC subjects (Fig. 1; Table 2). Nor was there any difference between TS and NC subjects in any other brain region (data not shown). There was nothing resembling a trend in differences of [^18^F]FEOBV binding between TS and NC subjects in any region. In TS subjects, there was no correlation between striatal [^18^F]FEOBV binding and global YGTSS scores: caudate, *r*^2^ = 0.002 (*p* = 0.88); putamen, *r*^2^ = 0.00 (*p* = 0.98).

**Figure:**
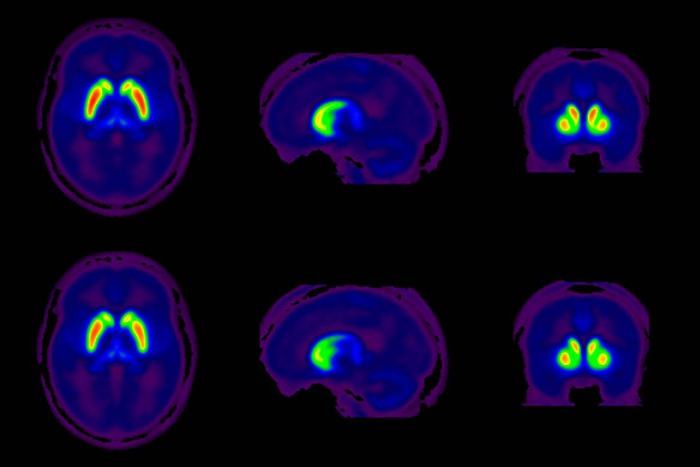
**Figure 1.** Striatal cholinergic terminal density measured by [^18^F]FEOBV PET in control (top row) and TS (bottom row) subjects. Averaged images from all subjects and images thresholded to optimize visualization of striatal [^18^F]FEOBV binding. No difference in striatal [^18^F]FEOBV binding between TS and NC subjects.

**Table 2.** Striatal [1**^8^F]FEOBV binding in TS and normal control subjects**

	Mean caudate BP (SD)	Mean putamen BP (SD)
TS (*N* = 12)	8.1016 (2.4500)	9.2805 (2.7901)
Normal control (*N* = 10)	7.8982 (0.9475)	9.1135 (1.0031)
TS % difference from control	102.6%	101.8%
*p*^1^	0.3974	0.4248

^1^ Two-tailed *t* test.

### MRI results

We assessed striatal volumes to exclude differences in striatal volumes between TS and control subjects as a confounding variable. There were no differences in caudate (*p* = 0.282) or putamen (*p* = 0.38) volumes between TS and controls subjects. TS mean caudate volume was 3895 mm^3^ (SD 781) and TS mean putamen volume was 6315 mm^3^ (SD 905). Control mean caudate volume was 4124 mm^3^ (SD 719) and control mean putamen volume was 6422 mm^3^ (SD 713).

## Discussion

Our results do not indicate any striatal cholinergic neuron deficit in TS. Our results do not even suggest any trend toward diminished striatal cholinergic terminals or, indeed, altered cholinergic projections in any brain region. These results differ markedly from those obtained in postmortem studies.

[^18^F]FEOBV is a highly specific ligand for VAChT, the protein responsible for pumping acetylcholine into synaptic vesicles. VAChT is expressed at uniquely high levels by cholinergic neurons and concentrated in cholinergic terminal synaptic vesicles. [^18^F]FEOBV binding appears to be a sensitive measure of cholinergic terminal integrity. In preclinical experiments, Parent et al. (2012, 2013) demonstrated that [^18^F]FEOBV PET imaging in rats detected modest (∼20%) changes in cortical cholinergic terminal density following basal forebrain lesions and smaller (∼10%) changes in hippocampal cholinergic innervation accompanying aging. While striatal cholinergic interneurons comprise only a small fraction (∼1%) of striatal neurons, they appear to have particularly luxurious axonal arbors. The expression of cholinergic neuron markers are higher in striatum than any other CNS region. This includes markers associated with cholinergic synapses and terminals such as acetylcholinesterase, VAChT, and the high affinity choline transporter (Vickroy et al., 1985). Cholinergic interneurons were historically thought to be the sole source of striatal cholinergic terminals. Using sensitive viral based tract tracing methods, Dautan et al. (2014) demonstrated that cholinergic pedunculopontine nucleus (PPN) complex neurons innervate the striatum. Prior work and their data suggest that pedunculopontine complex afferents make a relatively minor contribution to striatal cholinergic terminal density. It is possible, although unlikely, that an increase in PPN cholinergic afferent terminals could mask loss of striatal cholinergic interneurons. Striatal PPN cholinergic afferents, however, are collaterals of PPN neurons projecting to other regions such as the thalamus and substantia nigra. We found no evidence of increased cholinergic terminals in any brain region.

There are three major possibilities for the discrepancy between our results and the prior postmortem findings. One possibility is that the apparent deficit of striatal cholinergic interneurons described previously is an artifact secondary to agonal states and/or postmortem changes inevitable in postmortem human tissues. Kataoka et al. (2010) assessed striatal cholinergic interneuron density in TS and control subjects with two complementary probes. They measured the density of choline acetytransferase (CAT)-immunoreactive and large (24- to 42-μm soma diameter) calretinin-immunoreactive striatal neuron perikarya. It is possible that the expression of these proteins and/or their mRNAs is affected by agonal status. It is possible also that these proteins are degraded significantly after death. In the samples studied by Kataoka et al. (2010), there was a significant difference in postmortem interval between death and harvesting of TS and control tissues (TS, mean 22.6 h, range 11–30 h; control, mean 14.1 h, range 8.5–21 h; *p* < 0.0375, directional Mann–Whitney *U* test). Older biochemical literature on postmortem effects on brain CAT activity report varying results with some human and animal model studies describing modest postmortem effects on CAT activity and others describing significant declines in CAT activity with longer intervals between death and tissue freezing (Bowen et al., 1976; Fahn and Côté, 1976; Spokes and Koch, 1978; Spokes, 1979). Agonal state is known to differentially affect gene expression and may be important in this situation (Li et al., 2004). Spokes (1979), however, reported little effect of agonal state (sudden death versus prolonged illness) in humans on brain CAT activity.

Another possible reason for the discrepancy between our results and prior postmortem studies is medication effects. The subjects used in the study of Kataoka et al. (2010) are described as having severe TS and all were prescribed dopamine antagonist medications at the time of death. Some prior literature indicates that chronic dopamine antagonist administration in rodents reduces CAT activity and immunohistochemical detection of striatal cholinergic interneurons (Pedata et al., 1980; Mahadik et al., 1988; Terry et al., 2003; Kelley and Roberts, 2004). Only 1 of our TS subjects was using dopamine antagonists at the time of study. Whether dopamine antagonists affect the expression of calretinin in striatal neurons is unknown. It is possible that medication effects and any postmortem effects could have additive effects on identification of cholinergic striatal neurons.

A final possible explanation for discrepant imaging and postmortem results is disease heterogeneity. Tics are the defining feature of TS and it is plausible that like some other neurologic phenomena, tics could result from dysfunction in more than one region. The subjects used in the studies of Kalanithi et al. (2005), Kataoka et al. (2010), and Lennington et al. (2016) were selected partially on the basis of severe TS. Our subjects generally had milder phenotypes (Table 1). It is possible that a striatal cholinergic deficit could be associated with severity of tic expression and be characteristic of a subpopulation of TS subjects.

An unlikely cause of discrepant results would be dissociation between VAChT and CAT expression. Expression of these proteins is usually coordinated as VAChT by encoded within the first intron of the CAT gene. Differential processing or degradation of VAChT and CAT transcripts is possible but in their study of striatal transcriptomic changes in TS, Lennington et al. (2016) reported both diminished CAT and VAChT transcript expression, which is more consistent with agonal/postmortem and/or medication effect as causes of discrepant imaging and postmortem analysis results.

Our results have implications for efforts to model TS. Xu et al. (2015) reported that selective depletion of murine dorsolateral striatal cholinergic interneurons resulted in increased stress- and amphetamine-induced stereotypies. Martos et al. (2017) recently described a murine model with selective deletion of all dorsal striatal cholinergic interneurons, reporting compulsive social behaviors. Rapanelli et al. (2017) reported that joint depletion of dorsal striatal cholinergic and parvalbumin-immunoreactive interneurons produced increased stereotyped behaviors and social interaction deficits in male, but not female, mice. Our results are most consistent with preservation of striatal cholinergic integrity in TS and suggest that these murine models, and possibly, these rodent measures of motor and social dysfunction, are not relevant to TS.

While we do not find evidence of striatal cholinergic interneuron depletion in TS, cholinergic interneurons play a critical role in striatal function and striatal cholinergic signaling is a plausible target for pharmacologic intervention in all disorders with striatal dysfunction (Deffains and Bergman, 2015). Small clinical series and case reports suggest beneficial effects of cholinesterase inhibitors in TS (Niederhofer, 2006; Cubo et al., 2008). Given the risks of dopamine antagonist treatments, pharmacologic manipulation of striatal cholinergic neurotransmission is still a viable clinical research avenue in TS.

## Synthesis

Reviewing Editor: Cheryl Sisk, Michigan State University Decisions are customarily a result of the Reviewing Editor and the peer reviewers coming together and discussing their recommendations until a consensus is reached. When revisions are invited, a fact-based synthesis statement explaining their decision and outlining what is needed to prepare a revision will be listed below. The following reviewer(s) agreed to reveal their identity: Hitoshi Inokawa, Andreas Hartmann

Review synthesis:

This manuscript demonstrates normal expression of vesicular acetylcholine transporter in Tourette syndrome patients using a [18F]FEOBV PET. This is a well written, thoughtful manuscript regarding a major question in TS etiology, i.e. a potential default in cholinergic interneuron migration to the striatum as posited by Vaccarino's group in two highly cited and influential papers investigating a small (n=3/5 ; 2005/2010) postmortem sample. Based on these results, several groups have engaged in modeling these deficits in rodents, indeed identifying motor and behavioral abnormalities, although the relevance to tics in particular and TS in general is debatable (as always in these models). The interneuron migration deficit in TS has been investigated in other neurodevelopmental disorders (schizophrenia, ADHD) with interesting data and is quite elegant. However, reviewers agree that the results of the present paper, which call this model into question, are solid. The PET marker seems to be adequate and the controls reasonably well matched.

Please respond to the reviewers' comments below and revise your manuscript as appropriate.

1. As the authors note themselves, the severity of the patient population at hand might have been insufficient. Also, the n (12/10) is really small (but so were Vaccarino's although, obviously, acquiring postmortem tissue from TS patients is a whole different ballgame than scanning live patients). Please comment on the marker's sensitivity, especially provide more information on what it actually measures in the striatum: is the entire signal derived from cholinergic interneurons (which are not that abundant to begin with), or would cholinergic afferents from other brain regions contribute to the signal? With regard to GABA, it would be quite clear that the interneuron signal would be drowned in the MSN signal, for instance, although these are resident neuronal populations.

2. In lines 198-208, the authors cite two papers that support the idea that loss of cholinergic neurons cause behavioral phenotypes related to TS. In contrast, the results of this paper demonstrate normal expression of vesicular acetylcholine transporter, suggesting normal cholinergic neurons in TS. Thus, the authors claim that loss of cholinergic neurons may not be relevant to TS. The authors go on to cite Pappas et al. (2015) as a supporting paper for their claim. However, Pappas et al. (2015) shows that loss of cholinergic neurons causes a dystonic-like phenotype. So, I cannot follow the logic of the authors claim. Please provide further explanation.

3. I strongly disagree with the conclusions in the final paragraph. Although the present results are well worth publishing, I feel that here, the authors throw out the baby with tub water. First, no one claims (at least to my knowledge) that striatal cholinergic deficits are sufficient to cause tics in themselves; however, combined with other abnormalities, say GABAergic dysfunction, they may well be. Also, modulating cholinergic neurotransmission is an attractive and feasible target and actually, a few small clinical studies suggest that AchE inhibitors may be able to reduce tics, albeit with considerable side effects. Thus, because TS clinicians are still on the lookout for alternatives to antipsychotics, I would urge further exploration of the cholinergic interneurons hypothesis in TS; for instance, using the approach used in this paper with higher n (expensive, I know) and more severe disease phenotypes.

4. Authors should explicitly state whether informed consent from study participants was obtained or not.

5. Is there any relationship between severity of Tourette syndrome and PET signal? Because specimens of previous post-mortem studies were from older patients compared with this study, it may be a factor in the discrepant results.

6. Lines 128-30: authors refer to two-way t-tests. Do the authors mean paired t-tests? I don't know of a two-way t-test.

7. Please correct these typos: 1) line 62-63, delete “set” after (N=9); 2) lines 122 and 126, FreeSurfer vs Free-Surfer?
